# How Metabolic Diseases Impact the Use of Antimicrobials: A Formal Demonstration in the Field of Veterinary Medicine

**DOI:** 10.1371/journal.pone.0164200

**Published:** 2016-10-07

**Authors:** Didier Raboisson, Maxime Barbier, Elise Maigné

**Affiliations:** 1 IHAP, Université de Toulouse, INRA, ENVT, Toulouse, France; 2 ODR, INRA, Toulouse, France; National Research Council of Italy, ITALY

## Abstract

Decreasing the use of antimicrobials has become a primary objective for both human and veterinary medicine in many countries. Medical prevention and good nutrition are seen as key parameters for reducing antimicrobial use. However, little consideration has been given to how metabolic diseases may influence the use of antimicrobials in humans and animals through limiting the prevalence and severity of infectious diseases. To quantify this relationship using the example of a common metabolic disease in dairy cows (subclinical ketosis, SCK), we constructed a stochastic model reporting the total quantity of curative antimicrobials for a given population with the prevalence of cows at risk for SCK. We considered the prevalence of SCK, the relative risk of the disease in cases of SCK compared to no SCK and the use of antimicrobials to treat SCK-induced infectious diseases. Reducing the percentage of cows at risk for SCK from 80% to 10% was associated with an average decrease in the use of antimicrobials of 11% (prevalence of SCK from 34% to 17%, respectively) or 25% (prevalence of SCK from 68% to 22%, respectively), depending on the relative risk to contract SCK if risk was present. For a large percentage of the cows at risk for SCK, using a preventive bolus of monensin reduced the use of curative antimicrobials to the same level that was observed when the percentage of cows at risk for SCK was low. The present work suggests similar approaches for obesity and diabetes.

## Introduction

The use of antimicrobials in both human and veterinary medicine has caused a proliferation of resistant pathogens. The use of similar antimicrobials and the frequency of direct or indirect contacts between animals and humans has led to antimicrobial resistance (AMR) throughout both groups. Several strategies have been proposed to address the global issue of AMR, including: (i) implementing changes to promote research and development of new antimicrobials [1], (ii) reducing inappropriate antimicrobial use in instances of viral infection [2,3], and (iii) promoting the prevention of disease through vaccination. Improvements in communication, guidelines, diagnosis, and persuasive national plans are growing worldwide [4,5]. Antimicrobials are widely used in veterinary medicine; however, their use can vary depending on the country, species and context of production. Increasing societal pressure to promote sustainable use of antibiotics in veterinary medicine has led to changes in prescribing practices in many countries. For example, the French swine industry has spontaneously proposed a moratorium on the use of third and fourth generation cephalosporins, which has led to a continuous decrease in their use [6]. In addition, national plans have been implemented in several countries. The Netherlands reduced the use of antibiotics in veterinary medicine by 50% in 3 years [7]. France aims to reduce the use of antibiotics in animals by 25% in 5 years (http://agriculture.gouv.fr/le-plan-daction-ecoantibio-2012-2017). The initial results of this plan showed that the use of oral antibiotics were the first to be reduced in large proportions [6]. Reducing the use of antimicrobials in veterinary medicine is based on the same principles as human medicine: improving the prevention of infectious diseases, promoting diagnosis, banning or reducing the use of antimicrobials for prevention purposes or as collective treatments, and banning or reducing the use of antimicrobials for growth promotion (although this practice is already banned in many countries). Depending on the country and on the strength of the national animal health organisation, improvements in these practices are expected to occur quickly.

At the farm or animal level, strategies to reduce the use of antimicrobials are mainly focused on the prevention of endemic infectious diseases. Although alternatives to antimicrobials to treat illnesses in animals are also emerging, the main focus is to adopt good management practices to lower the prevalence of infectious diseases [8]. Prevention through vaccination is very efficient for some diseases, such as viral and bacterial diseases, and clinical protection is close to 100% for many of these diseases. However, vaccines only reduce the risk and severity of the clinical signs for many other diseases. Breeding practices, including housing conditions and animal handling, are key factors for the control of disease. Poor nutrition in animals also promotes infectious diseases through reduced immunity [9], and appropriate feeding practices are recognised as a way to prevent several disorders and infectious diseases. However, the increased demands on animal production, the competition in the international markets and the breeding of animals in groups has led to difficulties fulfilling the needs of individual animals. Although medical prevention has always been considered a key to reducing the use of antimicrobials, little consideration has been given to how metabolic diseases may influence antimicrobial use in both humans and animals through limiting the prevalence and severity of infectious diseases. Reducing the prevalence and improving the management of metabolic diseases may be two important strategies to address the use of antimicrobials in modern or developing societies. Quantifying the relationships between the total reduction in antimicrobial use in a population with different prevalences for a given metabolic disease is of great interest for public decision-making and allocation of resources. Here, we use the example of a common metabolic disease in the dairy industry to illustrate the strength of this relationship.

Subclinical ketosis (SCK) is a common metabolic disorder in dairy cows caused by dietary negative energy balance around or after calving. A certain degree of negative energy balance is expected in cows in late gestation and early lactation, but if the concentration of blood ketones increases dramatically, SCK can occur [10]. As a result of the imbalance between energy expenditure and ingestion, SCK is not only seen in high producing dairy cows but also in cows with moderate milk production and inadequate ingestion or feeding around calving. An equivalent disorder (pregnancy toxaemia) is seen in ewes before the birth of lambs in cases of high prolificacy. The lactational prevalence of SCK in Europe is estimated to be between 25% to 47% [11,12], and up to 85% of herds are reported to have an intra-herd prevalence of more than 25% [13]. SCK is associated with increased risk for most of the infectious diseases observed in cows during the early postpartum period (puerperal metritis, purulent vaginal discharge, placental retention, abomasum displacement, mastitis, lame) [14]. The treatment of these diseases most often includes antimicrobials. Prevention of SCK is based on adequate nutrition of the dairy cow during the dry period (before calving) and the most important risk factor during this period is overfeeding, which leads to fat cows at calving and reduced dietary intake around calving and early postpartum [10,15]. Until recently in Europe, there was no diet-based preventive solution for cows that were fat a few weeks prepartum, and weight reduction at this stage increases the risk of developing SCK. Recently, an oral-route bolus of monensin was authorised in Europe to prevent SCK when administered 3 weeks prior to calving in cows at risk for SCK. This preventative treatment strategy is expected to reduce the use of antimicrobials during lactation by preventing the development of SCK.

Therefore, the aim of the present study was to define the expected decrease in antimicrobial use through preventing the occurrence of a common metabolic disease in dairy cows (subclinical ketosis) using two strategies in the field: diet management or monensin bolus. We showed that preventing metabolic disease is a powerful way to decrease the use of antimicrobials and suggest that prevention of metabolic diseases such as obesity and diabetes can reduce the threat of AMR in humans.

## Materials and Methods

### Concept and model

**Fig 1:**
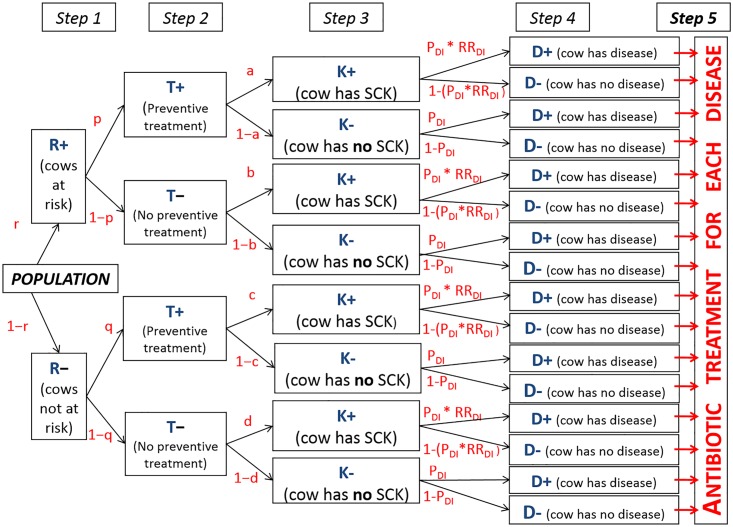
Conceptual flowchart. Flowchart representing the conceptual population with the various possible decisions made.

A conceptual model aims at simulating a given population and the various decisions made for different possible events, calculated in 5 successive steps (Fig 1). The population included (i) a subpopulation of cows at risk for SCK at a prevalence (r) and (ii) a subpopulation of cows not at risk for SCK at a prevalence (1-r) (Step 1). Additionally, farmers may use a preventive treatment (monensin bolus) on cows at risk for SCK with a probability (p) (Step 2). Because farmers do not make perfect decisions, they may also treat a cow not at risk for SCK with a risk (q). A situation without any preventive treatment monensin bolus is accounted for within the model with p = q = 0 (Fig 1). Then, the following denotations were made:

rp: the prevalence of cows at risk for SCK and preventively treated,
r*(1-p): the prevalence of cows at risk for SCK and not preventively treated,
(1-r)*q: the prevalence of cows not at risk for SCK and preventively treated,
(1-r)*(1-q): the prevalence of cows not at risk for SCK and not preventively treated.

The following step (Step 3) focused on the risk for a cow to actually develop SCK (Fig 1). The denotations used were:

a: the risk of SCK for a cow at risk of SCK but preventively treated,
b: the risk of SCK for a cow at risk of SCK and not preventively treated,
c: the risk of SCK for a cow not at risk of SCK but preventively treated,
d: the risk of SCK for a cow not at risk of SCK and not preventively treated.

The parameters b and d are related by Eq 1.

$$\text{b}=\text{d * }RR_{\text{SCK IF AT RISK}}$$ (1)

Where:

RR_SCK IF AT RISK_ is the relative risk of having SCK if its risk factors are present in a given cow.

The parameters a and b are related by Eq 2.

$$\text{a}=\text{b * }(1-EFF_{\text{MONENSIN}})$$ (2)

Where:

EFF_MONENSIN_ is the reduction in the prevalence of SCK due to the monensin bolus when administered to cows at risk for SCK.

The parameters d and a are related by Eq 3 as a result of Eqs 1 and 2.

$$\text{a}=\text{d * }RR_{\text{SCK IF AT RISK}}\text{ * }(1-EFF_{\text{MONENSIN}})$$ (3)

The parameter c was defined as d, meaning that the bolus has no efficacy on cows not at risk for SCK. The parameter d (risk of SCK for a cow not at risk for SCK and not preventively treated with monensin) represents the baseline “natural” risk of SCK in cows not exposed to risk factors. This value is rarely available as the prevalence of SCK (P_SCK_) reported in the literature often includes populations at risk and not at risk for SCK. The parameter d was then calculated from the literature using: (i) the prevalence of cows at risk for SCK (P_COWS AT RISK SCK_), (ii) P_SCK_ and (iii) RR_SCK IF AT RISK_ that were reported in each of the studies Eq (4).

$$\text{d}=\frac{P_{\text{COWS AT RISK SCK }}}{P_{\text{SCK }}*RR_{\text{SCK IF AT RISK}}+1-P_{\text{COWS AT RISK SCK}}}$$ (4)

The overall prevalence of SCK (P_SCK_) and the number of preventive treatments (N_TRT_) in the studied population were defined using Eqs 5 and 6.

$$P_{\text{SCK }}=\text{r * p * a }+\text{ r * }\left(1-\text{p}\right)\text{ * b }+\;\left(1-\text{r}\right)\text{ * q * c }+\;\left(1-\text{r}\right)\text{ * }\left(1-\text{q}\right)\text{ * d}$$ (5)

$$N_{\text{TRT }}=\text{ r * p }+\;\left(1-\text{r}\right)\text{ * q}$$ (6)

A targeting index (TI) was then defined according to Eq 7, which represents the ability of the decision maker to only treat cows at risk.

TI=r * p + (1−r) * q (7)

Step 4 focused on the risk of cows having an infectious disease (Fig 1). Cows with SCK have a higher risk for infectious diseases compared to cows without SCK Eq (8).

$$P_{{\text{DI}}_{i}\text{IF SCK }}=P_{{\text{DI}}_{i}\text{IF NO SCK }}*RR_{\text{DI}i\text{ IF SCK}}$$ (8)

Where:

$P_{{\text{DI}}_{i}\text{IF SCK }}$ the prevalence of disease (*i)* in cows with SCK,

_$P_{{\text{DI}}_{i}\text{IF NO SCK }}$_ the prevalence of disease (*i)* in cows without SCK,

*RR*_DI*i* IF SCK_ the relative risk of disease (*i)* in cows with SCK compared to without SCK.

Step 5 was to calculate the total quantity of curative antimicrobials (QTY_AM_CUR_) for a given population of cows as defined in Eqs 9 and 10. QTY_AM_CUR_ was the sum of the quantity of antimicrobials used for all the infectious diseases (*i)* for cows without SCK (left of the equation) or with SCK (right of the equation).

$$QTY_{\text{AM}\_\text{CUR }}=\Sigma_{1}^{D}\left(\left(1-P_{\text{SCK }}\right)\text{ * }P_{{\text{DI}}_{i}\text{IF NO SCK }}\text{*}QTY_{{\text{DI}}_{i}\;}\text{* }Coef_{\text{POND }}\;+\;P_{\text{SCK }}\text{ * }P_{{\text{DI}}_{i}\text{IF NO SCK }}\text{* }RR_{\text{DI}i\text{ IF SCK}}\text{ * }QTY_{{\text{DI}}_{i}}\text{* }Coef_{\text{POND }}\right)$$ (9)

Or

$$QTY_{\text{AM}\_\text{CUR }}=\Sigma_{1}^{D}(\;P_{{\text{DI}}_{i}\text{IF NO SCK }}\text{* }QTY_{{\text{DI}}_{i}\;}\text{* }Coef_{\text{POND}}\text{*}\left[\;P_{\text{SCK }}\text{* }RR_{{\text{DI}}_{i}\text{IF SCK}}+\left(1-P_{\text{SCK }}\right)\right]$$ (10)

Where:

$QTY_{{\text{DI}}_{i}\;}$: the quantity of antimicrobials used to treat one cow with disease (*i)*,

D: the number of infectious diseases (*i)*,

*Coef*_POND_: the coefficient of ponderation accounting for the percentage of ill cows actually treated with antimicrobials for the disease (*i*).

The total overall quantity of antimicrobials for a given population (QTY_AM_) was the sum of QTY_AM_CUR_ and the quantity of monensin, according to Eqs 11 and 12.

$$QTY_{\text{AM}}=QTY_{\text{AM}\_\text{CUR}}+\;QTY_{\text{AM}\_\text{MONENSIN}}$$ (11)

With:

$$QTY_{\text{AM}\_\text{MONENSIN}}=N_{\text{TRT}}*\;\text{Quantity of monensin within one bolus}$$ (12)

The model was run using Scilab (version 5.5.1) open source software (www.scilab.org) with 10,000 iterations, and the 95% prediction intervals (PI) and 95% confidence intervals (CIs) were calculated. The PI makes it possible to predict the situation for the next farm with 95% probability, and the CI indicates the situation in 95 of 100 farms visited.

### Calibration

The key input parameters of the models are summarised in Table 1. Most of the input parameters were included as a law of distribution (normal or log-normal) and not as a point estimate. The value of the parameter d was defined according to Eq 4 applied to results in the literature. Unfortunately, because the available studies did not report the 3 input parameters of Eq 4, the parameter d was applied to theoretical situations with P_COWS AT RISK SCK_ from 10 to 30%, P_SCK_ from 25% to 45% and RR_SCK IF AT RISK_ from 2 to 4 (S1 Table). The obtained value of d was approximately 15% in most of case and 10% in rare situations (Table 1). RR_SCK IF AT RISK_ was defined according to a meta-analysis [16] conducted on the outcomes “parity” and “body condition score” (BCS). Parity represents the number of previous calvings for a given cow and BCS represents the usual criteria to evaluate the fat deposits in cows [17]. BCS ranges from 1 to 5 points (given in ¼ point increments) and cows with a BCS between 3.75–4 points at calving are considered to be too fat and at risk for SCK [11,18,19]. The literature included (S2 Table) and the methods, results and discussion (S1 Text) of the meta-analysis are detailed in the supplementary files. The results of the meta-analysis showed it was difficult to precisely define RR_SCK IF AT RISK_. Two scenarios were then proposed, with RR_SCK IF AT RISK_ defined through LN(0.76,0.60) or LN(1.50,0.62) (Table 1). EFF_MONENSIN_ was evaluated from the report for the monensin bolus of the Committee for Medicinal Products for Veterinary Use from the European Medicines Agency (www.ema.europa.eu). Three other studies dealing with monensin in the form of a bolus or mixed in food were also included; however, the available data in the literature were limited compared to the Committee for Veterinary Medicinal Products data to define EFF_MONENSIN_ (S3 Table). Therefore, three values of EFF_MONENSIN_ were retained (Table 1). P_DI*i* IF NO SCK_ and RR_DI*i* IF SCK_ were defined by the meta-analysis and the literature review, and Coef_POND_ was defined from an experts’ opinion (S4 Table). The details of the methods and results were previously published [14,20]. All cows were treated with the same antibiotic in both scenarios (cephalosporin in scenario C or penicillin A in scenario P). The quantity of antibiotic injected for each treatment (QTY_DI*i*_) was defined according to the reglementary recommendations (http://www.med-vet.fr/) for a 600 kg (BW) dairy cow. We utilised 2 commonly used drugs in the dairy industry in France according to the authors’ expertise. For scenario C, 255 mg of cefquinome (Cobactan LC^®^, 3 syringes per treatment) and *in toto* 750 mg of amoxicillin and clavulanic acid was used in scenario P (Synulox^®^, 3 syringes per treatment) when administered through the intra-mammary route. Conversely, 3,000 mg of ceftiofur was used in scenario C (Excenel RTU^®^, 5 days of treatment), and 26,250 mg of amoxicillin and clavulanic acid was administered in scenario P (Synulox suspension^®^, 5 days of treatment) when given parenterally. The QTY_AM_MONENSIN_ was 31,828 mg of monensin (Kexxtone^®^), according the Medicinal Products for Veterinary Use report. All the models were run for an average 100-head dairy cow herd.

**Table 1:** Input parameters for the model.

	Law^1^	Main scenario	Sensitivity analysis	Details and references
d	N	0.15 (0.04) ^2^	0.10 (0.03) ^2^	S1 Table
OR_SCK IF AT RISK_	LN	0.76 (0.60) ^3^	1.50 (0.62) ^3^	S2 Table and S1 Text
EFF_MONENSIN_	//	0.66	0.45 & 0.85	S3 Table
P_DI IF NO SCK_, RR_DI IF SCK,_	N or LN	Average	Low and high	S4 Table, Raboisson 2014 2015
Coef_POND_	//	Average fixed		S4 Table
Curative antimicrobial		Cephalosporin	Penicillin A	

^1^: LN = LogNormal, N = Normal;

^2^: mean (and SD);

^3^: corresponding to RR_SCK IF AT RISK_ = 2 and 4.5

## Results

**Fig 2:**
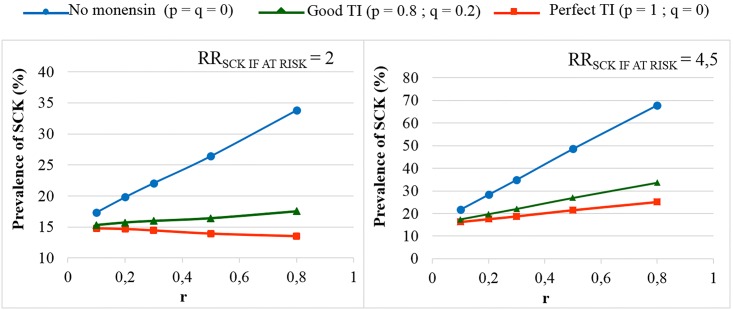
The prevalence of SCK for different proportions of cows at risk (r). The mean values are presented for the main scenario for two values of RR_SCK IF AT RISK_ and 3 situations of monensin use: no use, good targeting index (TI; 20% error rate in targeting cows) and perfect TI (no error in targeting cows) where p is the proportion of cows at risk for SCK that have been preventively treated with monensin and q is the proportion of cows not at risk for SCK that have been preventively treated with monensin.

**Fig 3:**
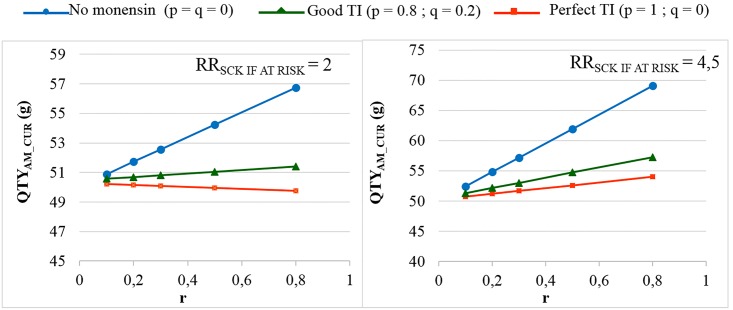
The quantity of curative antimicrobials used (QTY_AM_CUR_) for the different proportions of cows at risk (r). The mean values are presented for the main scenario for two values of RR_SCK IF AT RISK_ and 3 situations of monensin use: no use, good targeting index (TI: 20% error rate in targeting cows) and perfect TI (no error in targeting cows), where p is the proportion of cows at risk for SCK that have been preventively treated with monensin and q is the proportion of cows not at risk for SCK that have been preventively treated with monensin.

**Fig 4:**
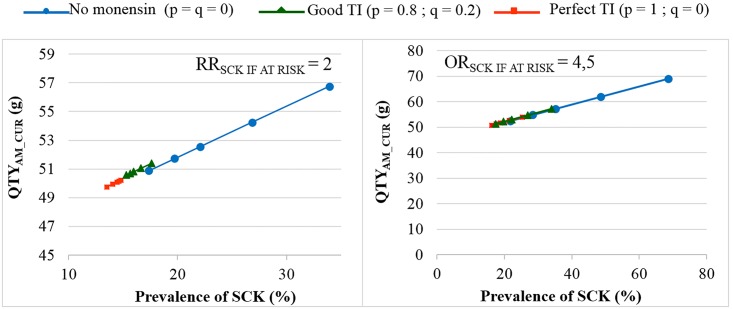
The quantity of curative antimicrobials used (QTY_AM_CUR_) for the various prevalences of SCK. The mean values are presented for the main scenario for two values of RR_SCK IF AT RISK_ and 3 situations of monensin use: no use, good targeting index (TI: 20% error rate in targeting cows) and perfect TI (no error in targeting cows), where p is the proportion of cows at risk for SCK that have been preventively treated with monensin and q is the proportion of cows not at risk for SCK that have been preventively treated with monensin.

In the main scenario, without any preventive medical treatment (p = q = 0), the average P_SCK_ decreased from 33.8% to 17.2% (OR_SCK IF AT RISK_ = 2) and from 67.9% to 21.7% (OR_SCK IF AT RISK_ = 4.5) when r decreased from 0.8 to 0.1 (Fig 2). Using the monensin bolus in a perfect way (TI = 1, or p = 1, q = 0) or in a correct way (p = 0.8 and q = 0.2) led to the stabilisation of P_SCK_ around the level observed for low values of r, whatever the initial value of r. Additionally, P_SCK_ decreased slightly for a perfect TI and a RR_SCK IF AT RISK_ = 2. The QTY_AM_CUR_ ranged from 44 g to 100 g when considering all possible combinations of calibration; however, the QTY_AM_MONENSIN_ ranged from 370 g to 2,600 g (S1 Fig), which led us to focus on the QTY_AM_CUR_ only in the remainder of the analyses. The changes in the QTY_AM_CUR_ (i) when r decreased, (ii) for no monensin bolus and (iii) for various TI values (Fig 3) followed a similar pattern as the changes observed for P_SCK_ (Fig 2). The average decrease in the QTY_AM_CUR_ with a decrease in r from 0.8 to 0.1 (P_SCK_ decreased from 33.8% to 17.2%, respectively) without any preventive treatment (p = q = 0) was 11% for a RR_SCK IF AT RISK_ = 2 (Figs 3 and 4). This increased to 25% for a RR_SCK IF AT RISK_ = 4.5 (P_SCK_ decreased from 67.9% to 21.7%, respectively). The QTY_AM_CUR_ was at the same order of magnitude (i) for a high r and a correct or perfect use of the monensin bolus and (ii) for a low r and any value of p and q (in accordance with the stabilisation of P_SCK_ around the level observed for a low r when r is high and the monensin bolus is correctly or perfectly used).

**Fig 5:**
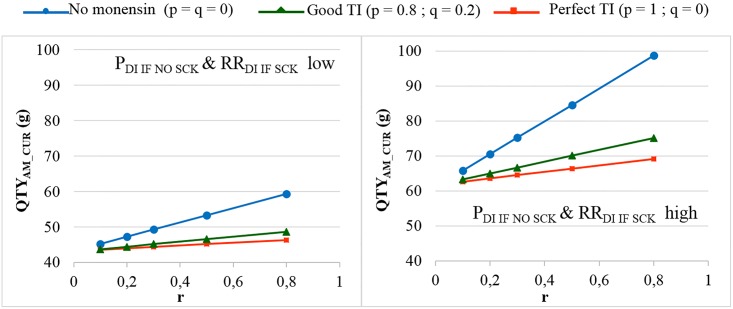
The quantity of curative antimicrobials used (QTY_AM_CUR_) for the different proportions of cows at risk (r) when RR_SCK IF AT RISK_ = 4.5. The mean values are presented for the main scenario for two values of RR_SCK IF AT RISK_ and 3 situations of monensin use: no use, good targeting index (TI: 20% error rate in targeting cows) and perfect TI (no error in targeting cows), where p is the proportion of cows at risk for SCK that have been preventively treated with monensin and q is the proportion of cows not at risk for SCK that have been preventively treated with monensin.

**Fig 6:**
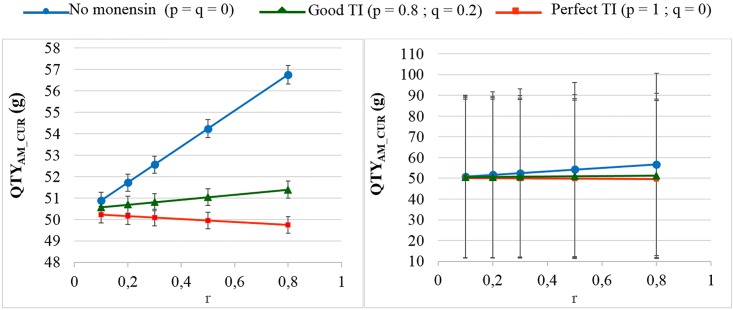
The quantity of curative antimicrobials used (QTY_AM_CUR_) for the different proportions of cows at risk (r) when RR_SCK IF AT RISK_ = 2. The mean values are presented with their 95% confidence intervals (left) and 95% prediction intervals (right) for the main scenario and for 3 situations of monensin use: no use, good targeting index (TI: 20% error rate in targeting cows) and perfect TI (no error in targeting cows), where p is the proportion of cows at risk for SCK that have been preventively treated with monensin and q is the proportion of cows not at risk for SCK that have been preventively treated with monensin.

The sensitivity analysis focused on EFF_MONENSIN,_ P_DI IF NO SCK_, RR_DI IF SCK_ and d. As expected, the results were not sensitive to EFF_MONENSIN_ in cases of no monensin treatment (S2 Fig). The stabilisation of P_SCK_ and the QTY_AM_CUR_ around the level observed for a low r when r is high and the monensin bolus if correctly or perfectly used was more or less important when EFF_MONENSIN_ changed. For an EFF_MONENSIN_ = 0.85 and a RR_SCK IF AT RISK_ = 2, a decrease in the QTY_AM_CUR_ when r increased was observed (S2 Fig). The high and low scenarios for P_DI IF NO SCK_ and RR_DI IF SCK_ significantly impacted the QTY_AM_CUR_ (Fig 5 and S3 Fig). The reduction in the QTY_AM_CUR_ permitted by good management practices (from a high r to a low r without the monensin bolus in both cases) increased to 20% (OR_SCK IF AT RISK_ = 2) and 40% (OR_SCK IF AT RISK_ = 4.5), respectively. Excluding the antibiotics administered through the intra-mammary route did not change the results (S4 Fig). The 95% CI ranges for all the results were very narrow, but the 95% PI ranges showed there was no possibility to predict the expected changes in the QTY_AM_CUR_ for the next population of 100 heads (Fig 6).

## Discussion

### Preventing metabolic diseases to reduce the use of antimicrobials

The present work quantifies the decrease in the use of antimicrobials through the prevention of a non-infectious metabolic disease in dairy cows. This decrease is expected and the stake of the present work was to quantify this relationship in a scenario without any preventive treatment (p = q = 0) and in a scenario with the use of a medical preventative treatment (p or q ≠ 0).

The decrease in the use of curative antimicrobials was lower than the decrease in the prevalence of the metabolic disease we observed when the risk factors of the metabolic disease disappeared (p = q = 0). The mean decrease in the use of curative antimicrobials was 11% and 25% for decreases in P_SCK_ of 50% (from 33.8% to 17.2%) and 70% (from 67.9% to 21.7%), respectively. This is in accordance with the base prevalence of the disease in cows without SCK (P_DI IF NO SCK_). However, this decrease in antimicrobial use, even if limited, is of interest to the medical community especially when one considers that this change was only observed for one disorder (although it is a key disease in dairy herds) and the expected decrease in antimicrobial use is in accordance with the decrease anticipated through other means. For example, the current French plan to reduce the use of antimicrobials in veterinary medicine aims for a 25% decrease in 5 years (http://agriculture.gouv.fr/le-plan-daction-ecoantibio-2012-2017). Despite efforts to change routines, the prescribing of antimicrobials for viral disorders is still a common practice reported in human medicine [21]. The mean decrease in the use of antimicrobials from 11% to 25% observed in the present work is trustworthy (see discussion of methods) because the P_SCK_ in the analysed situations is in the range of values for diagnoses made in the field and reported in the literature [11–13]. The extrapolation of the present results to human metabolic diseases such as diabetes is not possible at this stage, but the results suggests a need to consider prevention of obesity and diabetes as a means to address the issue of AMR.

The present work highlights the importance of substitution between the use of monensin and other critical antibiotics on dairy farms. The financial implications of substituting different medical treatments in livestock breeding are well described [8], particularly for the prevention of diseases with vaccines and the treatment of diseases (if they occur) with last-generation antimicrobials. The present work questions the substitution of monensin (administered orally) for critical antibiotics (administered parenterally). A bolus of monensin (administered orally) was reported to induce phenotypically expressed but not genetically stable resistance [22,23]. Therefore, whether we should recommend the prescription of old-generation antimicrobials to prevent the development of AMR with new antimicrobials remains a key question for microbiologists and epidemiologists in both human and veterinary medicine.

### Methods and calibration of the models

The present work put stress on a robust calibration. First, the 95% PI showed that it was difficult to predict the expected decrease in the curative use of antimicrobials for a 100-head population in which changes in routines led to change in the values of r and P_SCK_. These predictions are not yet available. Conversely, the expected decrease in the curative use of antimicrobials is precisely defined for a large population, as was evident by the 95% CI. This average effect is relevant because the mean change in the use of antimicrobials, and not the changes observed in extreme situations, is of interest when considering AMR. Additionally, the values of P_SCK_ in the present work agree with the values observed in the field [11–13]. This demonstrates the correct calibration of the model, despite the extreme range of values for the parameter r, which may falsely suggest that only the extreme situations were considered and compared. Several efforts were made to accurately estimate the different input parameters in the present study, including a previous meta-analysis [14], a review of the parameters [20] and complementary work performed for this study (S1 text). Moreover, the value of the parameter d highly influences the model and must be determined accurately. Furthermore, the values of a, b and c directly depended on the value of d. Two recent studies reported the percentages of cows not exposed to the related risk factors for SCK (30 to 40%), but these values were considered out of the average expected range in accordance with the mean P_SCK_ of these 2 studies (50%) [11,18]. The values of d were consequently defined according to Eq (4) and S1 Table. Simulations performed with d = N(0.1, 0.03) instead of d = N(0.15;0.04) showed a slightly lower decrease in the QTY_AM_CUR_ when the value of r decreased, without changing the range of the QTY_AM_CUR_ values. Finally, the value for the percentage of cows with a given disorder actually treated with antimicrobials (Coef_POND_) was defined in accordance with expert opinions. These coefficients may change slightly between farms, practitioners and livestock systems, but they remain fixed for the current study and did not influence the percentage of decrease in the use of antimicrobials observed when the metabolic disease prevalence decreased. Similarly, considering another curative antimicrobials instead of cephalosporin changed the absolute values of the QTY_AM_CUR_ but did not change the percentage of decrease in the QTY_AM_CUR_, as observed with simulations conducted with penicillin A. Many veterinarians prescribe antimicrobials other than cephalosporin to dairy cows; however, cephalosporin was a common antimicrobial for the treatment of sick dairy cows in the field in Europe.

## Conclusions

The present work highlights the importance of managing metabolic diseases to reduce the use of antimicrobials by examining a common veterinary example (SCK) in dairy cows. Decreasing the incidence of the metabolic disease decreased the use of antimicrobials up to 40%, and the average decrease was close to the 25% decrease objective described in the French veterinary plan for antimicrobial decrease. The results also suggest to analyse how prevention of obesity and diabetes may help to address the issue of AMR.

## Supporting Information

S1 Fig The overall quantity of antimicrobials used (QTY_AM_) for the different proportions of cows at risk (r). (PDF)

S2 Fig The quantity of curative antimicrobials used (QTY_AM_CUR_) for the different proportions of cows at risk (r). (PDF)

S3 Fig The quantity of curative antimicrobials used (QTY_AM_CUR_) for the different proportions of cows at risk (r) when RR_SCK IF AT RISK_ = 2. (PDF)

S4 Fig The overall quantity of antimicrobials used (QTY_AM_) and the quantity of curative antimicrobials used (QTY_AM_CUR_) for the different proportions of cows at risk (r) when RR_SCK IF AT RISK_ = 4.5. (PDF)

S1 Table The theoretical definition of the value of parameter d. (PDF)

S2 Table The raw data used to calculate the relative risk of having SCK for cows at risk for SCK (RR_SCK IF AT RISK_). (PDF)

S3 Table The raw data used to calculate the efficacy of the monensin bolus (EFF_MONENSIN_) to prevent subclinical ketosis (SCK). (PDF)

S4 Table The input parameters for the model. (PDF)

S1 Text The detailed methods, results and discussion of the meta-regressions performed to define the value of RR_SCK IF AT RISK_. (PDF)
