# Supplementary material for: How Metabolic Diseases Impact the Use of Antimicrobials: A Formal Demonstration in the Field of Veterinary Medicine
Source: PLoS One. 2016 Oct 7;11(10):e0164200. doi: 10.1371/journal.pone.0164200 (PMC5055344; doi:10.1371/journal.pone.0164200)
Supplement: S4 Fig — (PDF) [file pone.0164200.s004.pdf]

S4 Fig. The overall quantity of antimicrobials used (QTY_{AM}) and the quantity of curative antimicrobials used (QTY_{AM_CUR}) for the different proportions of cows at risk (r) when $RR_{SCK\ IF\ AT\ RISK} = 2$.

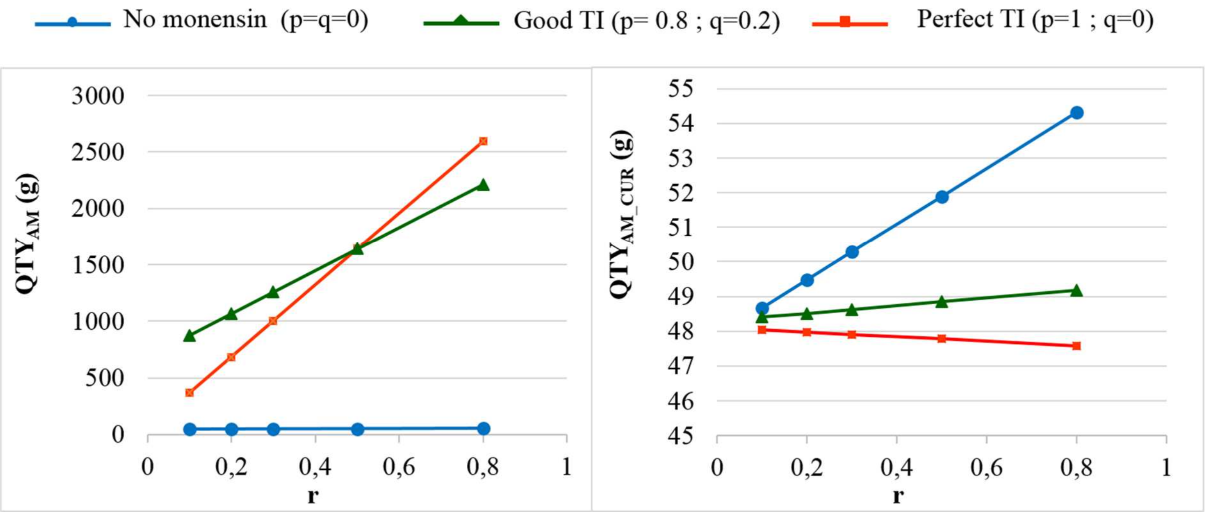

The mean values are presented for 3 situations of monensin use: no use, good targeting index (TI), meaning 20% of errors in targeting cows and perfect targeting index (no error in targeting). p was the proportion of cows at risk of SCK that have been treated preventively with monensin, and q was the proportion of cows not at risk of SCK that have been treated preventively with monensin.
