# Supplementary material for: How Metabolic Diseases Impact the Use of Antimicrobials: A Formal Demonstration in the Field of Veterinary Medicine
Source: PLoS One. 2016 Oct 7;11(10):e0164200. doi: 10.1371/journal.pone.0164200 (PMC5055344; doi:10.1371/journal.pone.0164200)
Supplement: S1 Table — (PDF) [file pone.0164200.s005.pdf]

S1 Table. The theoric definition of the value of parameter d.

$P_{COWS\ AT\ RISK\ SCK}$	P_{SCK}	$RR_{SCK\ IF\ AT\ RISK}$	Value of d obtained[1]	$P_{COWS\ AT\ RISK\ SCK}$	P_{SCK}	$RR_{SCK\ IF\ AT\ RISK}$	Value of d obtained[1]
0.2	0.25	2	0.154	0.1	0.40	3	0.048
0.2	0.30	2	0.143	0.1	0.45	3	0.044
0.2	0.35	2	0.133	0.1	0.50	3	0.042
0.2	0.40	2	0.125	0.3	0.25	3	0.207
0.2	0.45	2	0.118	0.3	0.30	3	0.188
0.2	0.50	2	0.111	0.3	0.35	3	0.171
0.1	0.25	2	0.071	0.3	0.40	3	0.158
0.1	0.30	2	0.067	0.3	0.45	3	0.146
0.1	0.35	2	0.063	0.3	0.50	3	0.136
0.1	0.40	2	0.059	0.2	0.30	4	0.100
0.1	0.45	2	0.056	0.2	0.35	4	0.091
0.1	0.50	2	0.053	0.2	0.40	4	0.083
0.3	0.25	2	0.250	0.2	0.45	4	0.077
0.3	0.30	2	0.231	0.2	0.50	4	0.071
0.3	0.35	2	0.214	0.1	0.25	4	0.053
0.3	0.40	2	0.200	0.1	0.30	4	0.048
0.3	0.45	2	0.188	0.1	0.35	4	0.043
0.3	0.50	2	0.176	0.1	0.40	4	0.040
0.2	0.30	3	0.118	0.1	0.45	4	0.037
0.2	0.35	3	0.108	0.1	0.50	4	0.034
0.2	0.40	3	0.100	0.3	0.25	4	0.176
0.2	0.45	3	0.093	0.3	0.30	4	0.158
0.2	0.50	3	0.087	0.3	0.35	4	0.143
0.1	0.25	3	0.061	0.3	0.40	4	0.130
0.1	0.30	3	0.056	0.3	0.45	4	0.120
0.1	0.35	3	0.051	0.3	0.50	4	0.111

1: the parameter d was calculated here thanks to (i) the prevalence of cows at risk of SCK ($P_{COWS\ AT\ RISK\ SCK}$), (ii) P_{SCK} and (iii) $RR_{SCK\ IF\ AT\ RISK}$ as indicated above (Eq. 4. of the manuscript), considering several values of the 3 input parameters.

$$d = P_{COWS\ AT\ RISK\ SCK} / (P_{SCK} * RR_{SCK\ IF\ AT\ RISK} + 1 - P_{COWS\ AT\ RISK\ SCK})$$
