# Supplementary material for: How Metabolic Diseases Impact the Use of Antimicrobials: A Formal Demonstration in the Field of Veterinary Medicine
Source: PLoS One. 2016 Oct 7;11(10):e0164200. doi: 10.1371/journal.pone.0164200 (PMC5055344; doi:10.1371/journal.pone.0164200)
Supplement: S2 Table — (PDF) [file pone.0164200.s006.pdf]

S2 Table. The raw data used to calculate the relative risk of having SCK for cows at risk for SCK ($RR_{SCK\ IF\ AT\ RISK}$)

Outcome variable			Risk		Covariate			
Variable	Definition[1]	Prevalence[2]	RR[3]	Se[4]	Season	Parity[4]	BCS[4]	Reference
BCS[4]	≤ 3 / > 3		0.67	0.15	1	1	na	[1]
BCS	≥ 4 / < 4		1.58	0.17	1	1	na	[1]
BCS	3.25 to 3.75/≤3	61/22	1.5	0.14	1	1	na	[2]
BCS	≥ 4 / < 3	17/22	2.7	0.2	1	1	na	[2]
BCS	Moderate / low	44/32	1.1	0.07	0	1	na	[3]
BCS	High / low	24/32	1.2	0.08	0	1	na	[3]
Parity[4]	P3/P2		1.59	0.13	1	na	1	[1]
Parity	P3/P1		3.13	0.14	1	na	1	[1]
Parity	P2/P1	28/30	1.3	0.2	1	na	0	[4]
Parity	P3/P1	41/30	2.1	0.4	1	na	0	[4]
Parity	P2/P1	27/25	2.1	0.16	1	na	1	[2]
Parity	≥P3/P1	48/25	2.8	0.15	1	na	1	[2]
Parity	P2/P1		2.21	0.36	0	na	0	[5]
Parity	≥P3/P1		4.5	0.33	0	na	0	[5]
Parity	P2/P1		3.29	0.37	0	na	0	[5]
Parity	≥P3/P1		5.92	0.46	0	na	0	[5]
Parity	P2/P1	35/34	1.1	0.18	0	na	1	[3]
Parity	≥P3/P1	36/34	1.7	0.13	0	na	1	[3]

Na : not applicable ; 1: denoted “at risk condition” / “reference condition” ; 2: denoted “number of cows at risk” / “number of cows not at risk” for the outcome variable ; 3: Relative risk and standard error of the linked log normal law; 4:Parity represents the number of calvings for a given cow and BCS represents the usual criteria to evaluate the fat deposit cows (ref). BCS ranges from 1 to 5 points, is given at ¼of points and cows above 3.75- 4 points of BCS at calving at considered as too fat and at risk of SCK

REFERENCES

1. Duffield TF, Sandals D, Leslie KE, Lissemore K, McBride BW, et al. (1998) Efficacy of monensin for the prevention of subclinical ketosis in lactating dairy cows. J Dairy Sci 81: 2866-2873.
2. Vanholder T, Papen J, Bemers R, Vertenten G, Berge AC (2015) Risk factors for subclinical and clinical ketosis and association with production parameters in dairy cows in the Netherlands. J Dairy Sci 98: 880-888.
3. McArt JA, Nydam DV, Oetzel GR (2013) Dry period and parturient predictors of early lactation hyperketonemia in dairy cattle. J Dairy Sci 96: 198-209.
4. Berge AC, Vertenten G (2014) A field study to determine the prevalence, dairy herd management systems, and fresh cow clinical conditions associated with ketosis in western European dairy herds. J Dairy Sci 97: 2145-2154.
5. Lomander H, Gustafsson H, Svensson C, Ingvartsen KL, Frossling J (2012) Test accuracy of metabolic indicators in predicting decreased fertility in dairy cows. J Dairy Sci 95: 7086-7096.
