# Supplementary material for: How Metabolic Diseases Impact the Use of Antimicrobials: A Formal Demonstration in the Field of Veterinary Medicine
Source: PLoS One. 2016 Oct 7;11(10):e0164200. doi: 10.1371/journal.pone.0164200 (PMC5055344; doi:10.1371/journal.pone.0164200)
Supplement: S3 Table — (PDF) [file pone.0164200.s007.pdf]

S3 Table. The raw data used to calculate the efficacy of the monensin bolus ($EFF_{MONENSIN}$) to prevent subclinical ketosis (SCK).

Coefficient of reduction of the SCK prevalence	$EFF_{MONENSIN}$	Bolus of monensin/ Report submitted to authorities for the selling authorisation	Raw data	Reference
0.34	0.66	Yes / Yes	Trial 1, cumulated prevalence of SCK = 11.5 versus 25.6, based on *Least Square Mean*	[1]
0.27	0.63	Yes / Yes	Trial 2, cumulated prevalence of SCK = 3.3 versus 12.3, based on *Least Square Mean*	[1]
0.26	0.74	Yes / Yes	Trial 3, prevalence of SCK at day 2 postpartum = 8.2% versus 32.1% based on BHBA threshold to diagnose SCK of 1.0 mmol/L	[1]
0.15	0.85	Yes / Yes	Trial 4, prevalence of SCK at day 2 postpartum = 3.0% versus 19.6% based on BHBA threshold to diagnose SCK of 1.4 mmol/L	[1]
0.46	0.54	Yes / No	$EFF_{MONENSIN}$ calculated on the raw mean of SCK prevalence	[2]
0.80	0.20	No / No	$EFF_{MONENSIN}$ calculated on mean BHBA between exposed and control groups	[3]
0.55	0.45	Na / Na	$EFF_{MONENSIN}$ calculated on mean BHBA between exposed and control groups	[4]
0.55	0.45	Yes / No	$EFF_{MONENSIN}$ calculated on mean BHBA between exposed and control groups	[5]

Na: not available ; BHB: Beta-hydroxy butyrate

REFERENCES

1. CVMP (2012) CVMP assessment report for Kexxtone (EMEA/V/C/002235). www.ema.europa.eu.
2. Duffield TF, Sandals D, Leslie KE, Lissemore K, McBride BW, et al. (1998) Efficacy of monensin for the prevention of subclinical ketosis in lactating dairy cows. J Dairy Sci 81: 2866-2873.
3. Duffield TF, Sandals D, Leslie KE, Lissemore K, McBride BW, et al. (1998) Effect of prepartum administration of monensin in a controlled-release capsule on postpartum energy indicators in lactating dairy cows. J Dairy Sci 81: 2354-2361.
4. Sauer FD, Kramer JK, Cantwell WJ (1989) Antiketogenic effects of monensin in early lactation. J Dairy Sci 72: 436-442.
5. Thomas EE, Poe SE, R.K. M, D.H. M, Alrich RD (1993) Effect of feeding monensin to dairy cows on milk production and serum metabolite during early lactation Journal of Dairy Science 76(Suppl. 1): 280.
