# Supplementary material for: How Metabolic Diseases Impact the Use of Antimicrobials: A Formal Demonstration in the Field of Veterinary Medicine
Source: PLoS One. 2016 Oct 7;11(10):e0164200. doi: 10.1371/journal.pone.0164200 (PMC5055344; doi:10.1371/journal.pone.0164200)
Supplement: S4 Table — (PDF) [file pone.0164200.s008.pdf]

S4 Table. The input parameters for the model

	Baseline Scenario		Low Scenario		High Scenario	
$P_{DI\ IF\ NO\ SCK}$[1]	Law[1]		Law[1]		Law[1]	
Left abomasum displacement	N	2.0 (1.0)	N	2.0 (1.0)	N	4.1 (6.0)
Right abomasum displacement	N	0.7 (0.33)	N	0.7 (0.33)	N	1.4 (2.0)
Placental retention	N	8 (3)	N	8 (3)	N	8 (3)
Metritis	N	8 (8)	N	8 (8)	N	8 (8)
Purulent vaginal discharge	N	5.8 (3.3	N	5.8 (3.3)	N	5.8 (3.3)
Subclinical endometritis	N	15 (6.5)	N	15 (6.5)	N	15 (6.5)
Clinical mastitis	N	11 (6)		Ni	N	11 (6)
Lame	N	26 (25)		Ni	N	26 (25)
$RR_{DI\ IF\ SCK}$[2]						
Left abomasum displacement	LN	1.20 (0.12)	LN	1.20 (0.12)	N	5.2 (3.5)
Right abomasum displacement	LN	0.53 (0.73)	LN	0.53 (0.73)	N	1.6 (1.2)
Placental retention	N	1.60 (0.33)	N	1.60 (0.33)	N	1.60 (0.33)
Metritis	LN	0.56 (0.07)	LN	0.56 (0.07)	N	3.4 (4.3)
Purulent vaginal discharge	N	2.30 (0.70)	N	2.30 (0.70)	N	2.30 (0.70)
Subclinical endometritis	N	1.40 (0.30)	N	1.40 (0.30)	N	1.40 (0.30)
Clinical mastitis	N	1.64 (0.20)		Ni	N	1.64 (0.20)
Lame	N	2.0 (0.40)		Ni	N	2.0 (0.40)
$Coef_{POND}$[3]						
Left abomasum displacement	//	0.9				
Right abomasum displacement	//	1.0				
Clinical ketosis	//	0.0				
Placental retention	//	0.5				
Metritis	//	0.8				
Purulent vaginal discharge	//	0.1				
Subclinical endometritis	//	0				
Clinical mastitis	//	0.8				
Lame	//	0.05				

Ni: not included in this scenario; 1: LN=LogNormal, N=Normal; 2: According to [1,2] 3: Data were estimated by authors for each disease according to (ii) the severity and prognostic associated, (ii) the infectious component involved and (iii) the most common practices seen or guides of practices defined in literature (S5 Table), according to studies included in [1,2] were used.

REFERENCES

1. Raboisson D, Mounie M, Maigne E (2014) Diseases, reproductive performance, and changes in milk production associated with subclinical ketosis in dairy cows: a meta-analysis and review. J Dairy Sci 97: 7547-7563.
2. Raboisson D, Mounie M, Khenifar E, Maigne E (2015) The economic impact of subclinical ketosis at the farm level: Tackling the challenge of over-estimation due to multiple interactions. Prev Vet Med.
