# Supplementary material for: How Metabolic Diseases Impact the Use of Antimicrobials: A Formal Demonstration in the Field of Veterinary Medicine
Source: PLoS One. 2016 Oct 7;11(10):e0164200. doi: 10.1371/journal.pone.0164200 (PMC5055344; doi:10.1371/journal.pone.0164200)
Supplement: S1 Text — (PDF) [file pone.0164200.s009.pdf]

S1 Text. The detailed methods, results and discussion of the meta-regressions performed to define the value of $RR_{SCK\ IF\ AT\ RISK}$.

Methods

$RR_{SCK\ IF\ AT\ RISK}$ was defined thanks to a meta-analysis [1]. It was conducted on the outcomes "parity" and "Body Condition Score (BCS)" using the Metafor package of R (version 3.0.2; R Foundation for Statistical Computing, Vienna, Austria). The different steps were recently outlined [2]. Only the two risk factors of SCK parity and BCS were included in the present meta-analysis. Parity represents the number of previous calvings for a given cow and BCS represents the usual criteria to evaluate the fat deposit cows [3]. BCS ranges from 1 to 5 points, is given at ¼of points and cows above 3.75- 4 points of BCS at calving at considered as too fat and at risk of SCK [3-6].

Results and discussion

The association between SCK and parity was reported in 12 models from 5 publications, some of being adjusted on BCS (S2 Table). The heterogeneity of the dataset was high (I^2 = 78% [95% CI=55-94] and Q statistics χ^2 = 45, df = 11, $P < 0.001$). The intercept of the log-effect size in the mixed-effects model with a random effect publication was 0.79 (SE=0.12, P<0.001), which corresponded to an effect size of 2.22 (95% CI=1.72-2.84). This is the mean RR for parity > 1 compared to parity =1, as this definition was used in the included studies. Including the moderators parity reduced the heterogeneity by 53%, and showed show a RR of 2.82 [95%CI =1.87-4.10] for parity = 3 compared to parity = 2.

The association between SCK and parity was reported in 6 models from 3 publications, all adjusted on parity (S2 Table). The heterogeneity of the dataset was high (I^2 = 88% [95% CI=48-98] and Q statistics χ^2 = 40, df = 5, $P < 0.001$). The intercept of the log-effect size in the mixed-effects model with a random effect publication was 0.28 (SE=0.18, P<0.001), which corresponded to an effect size of 1.27 (95%CI=0.92-1.77). This is the mean RR for BCS > 3 compared to BCS< 3, as this definition was used in the included studies. Including the moderators BCS reduced the heterogeneity by 53%, and showed show a RR of 1.63 [95%CI =1.04-2.56] for NEC > 4 compared to NEC < 3.

The sensitivity analysis showed no outlier for the meta-regressions (data not shown). Based on these results (RR for risk parity and BCS of 2.82 and 1.63, respectively), two final scenarios were retained (Table 1). The first onE was defined as the average of the two main

risk factors, accounting that the raw models were at least for part adjusted by the other variable. The lognormal law (mean and se) LN(0.76,0.60) was then used for $RR_{SCK\ IF\ AT\ RISK}$ in the scenario 1 (corresponding to RR=2). The second scenario considered an additivity within these 2 risk factors, because of the only partially adjustment on the other variable of the raw data, and because other rarely reported risk factors were not included here. For instance, the variable twin birth is not statistically significant when added to parity and BCS to explain the risk of SCK [4]. Moreover the interaction between parity and BCS is only statistically significant in models using given definition of SCK and not with other definition of SCK [4]. The lognormal law (mean and se) LN(1.50,0.62) was then used for $RR_{SCK\ IF\ AT\ RISK}$ in the scenario 2 (corresponding to RR=4.5).

REFERENCES

1. Viechtbauer W (2010) Conducting Meta-Analyses in R with the metafor Package. Journal of Statistical Software 36: 1-48.
2. Raboisson D, Mounie M, Maigne E (2014) Diseases, reproductive performance, and changes in milk production associated with subclinical ketosis in dairy cows: a meta-analysis and review. J Dairy Sci 97: 7547-7563.
3. Edmonson AJ, Lean IJ, L.D. W, Farver T, G. W (1989) A Body Condition Scoring Chart for Holstein Dairy Cows. Journal of Dairy Science 72: 68-78.
4. McArt JA, Nydam DV, Oetzel GR (2013) Dry period and parturient predictors of early lactation hyperketonemia in dairy cattle. J Dairy Sci 96: 198-209.
5. Lomander H, Gustafsson H, Svensson C, Ingvartsen KL, Frossling J (2012) Test accuracy of metabolic indicators in predicting decreased fertility in dairy cows. J Dairy Sci 95: 7086-7096.
6. Vanholder T, Papen J, Bemers R, Vertenten G, Berge AC (2015) Risk factors for subclinical and clinical ketosis and association with production parameters in dairy cows in the Netherlands. J Dairy Sci 98: 880-888.
